# Study of sedative activity of different extracts of *Kaempferia galanga* in *Swiss albino* mice

**DOI:** 10.1186/s12906-015-0670-z

**Published:** 2015-05-27

**Authors:** Mohammad Shawkat Ali, Pritesh Ranjan Dash, Mahmuda Nasrin

**Affiliations:** Department of Pharmacy, BRAC University, 41, Pacific Tower, Mohakhali, Dhaka Bangladesh; Department of Pharmacy, Jahangirnagar University, Savar, Dhaka Bangladesh

**Keywords:** *Kaempferia galanga*, Zingiberaceae, Thiopental sodium induced sleeping time test, Hole cross test, Open field test

## Abstract

**Background:**

*Kaempferia galanga* is an important medicinal plant and has been traditionally used to help restlessness, stress, anxiety, depression etc. in tropics and subtropics of Asia including Bangladesh, India, China, Japan and Indochina. Literature survey revealed that there are very less reports on neuropharmacological activity of this plant. Therefore, the present study investigated the sedative activity of different extracts of rhizome and leaf of *Kaempferia galanga*.

**Methods:**

The sedative activity was evaluated by using thiopental sodium induced sleeping time, hole cross and open field tests in *Swiss albino* mice at the doses of 100 and 200 mg/kg body weight per oral (p.o). The acetone extract of rhizome (ACR), as well as petroleum ether fraction (PEF), chloroform fraction (CHF), methanol fraction (MEF) and acetone extract of leaf (ACL) were examined for sedative activity.

**Results:**

In the sedative activity study, all the extracts exhibited significant (p < 0.05 and p < 0.001) reduction of onset and duration of thiopental sodium induced sleeping time, reduction of locomotor and exploratory activities in the hole cross and open field tests. In thiopental sodium induced sleeping time test, the chloroform extract of rhizome (200 mg/kg) showed maximum 358.55 % effect in duration of loss of righting reflex, whereas the standard drug Diazepam (2 mg/kg) produced 231.42 % effect. In hole cross and open field tests, maximum 95.09 % and 95.58 % suppression of locomotor activity were observed with the acetonic leaf extract (200 mg/kg) whereas suppression of locomotor activity of the standard drug Diazepam were 71.70 % and 70.58 % respectively.

**Conclusion:**

The present study indicates that the acetone extracts of rhizome and leaf of *Kaempferia galanga* including fractions possess central nervous system (CNS) depressant properties which supports its use in traditional medicine. So, the plant may be further investigated to find out for its pharmacological active natural products.

## Background

*Kaempferia galanga* (Chandramulika in Bengali) belonging to the family Zingiberaceae is an aromatic perennial herb with tuberous rootstocks. It is cultivated throughout Southeast Asia including Bangladesh and also introduced into Northern Australia [1]. The rhizome of the plant finds an important place in indigenous medicine carminative, diuretic, aromatic stomachic, insecticidal and incense. In Bangladesh, rhizomes juices of *Kaempferia galanga* are used as a remedy for toothache or a wash for dandruff or scabs on the head. In China, this plant is used for hypertension, pectoral and abdominal pains, headache, toothache, rheumatism, dyspepsia, coughs and inflammatory tumor [2]. It also has a long history of fragrance use to help restlessness, stress, anxiety and depression. In Japan, *Kaempferia galanga* has been used for improving sleep or minimizing stressful situations [3]. Pharmacological properties such as anti-inflammatory and analgesic [4, 5], antidiarrhoeal [1], nematicidal [6], mosquito repellent and larvicidal [7–9], vasorelaxant [10], sedative [3], antineoplastic [11–13], antimicrobial [14–16], anti-oxidant [17, 18] and cytotoxic [16] activity has been reported. The rhizomes of this plant contain volatile oil and other important compounds of enormous medicinal values and they are very demanding to the traditional health care practitioner [1]. Most vital constituents ethyl-cinnamate and ethyl–*para*-methoxycinnamate has already been isolated from this plant [19, 20, 3]. Previous investigations on this plant suggested that the hexane extract of the plant showed the sedative activity. Therefore, as a part of our continuing studies [1, 2, 16, 21] on natural products for their pharmacological properties we investigated acetone extracts of different parts of the plant of *Kaempferia galanga* for their sedative activity.

## Methods

### Collection of the plant

The plant of *Kaempferia galanga* was collected from the local area of Mauoa, Dhaka during December 2011. The collected plant was then identified by Bushra Khan, Principal Scientific Officer, Bangladesh National Herbarium, Mirpur, Dhaka and a voucher specimen had been deposited (DACB: 36,064) for further reference.

### Extraction and fractionation of the plant material

The plant parts were extracted by a cold extraction method. The rhizome (900 g) and leaf (200 g) powder were taken and soaked with 2700 ml and 600 ml of acetone for 3 consecutive days at 25 °C. The extracts were filtered and evaporated on rotary evaporator under reduced pressure. Recovered solvent was again used for percolation for another 3 days. The process was repeated three times to obtain 58 g rhizome (yield 6.45 %) and 4.14 g leaf (yield 2.07 %) extract of *Kaempferia galanga*. The rhizome extract was further partitioned using petroether, chloroform and methanol. The acetone extract of rhizome (ACR), as well as petroleum ether fraction (PEF), chloroform fraction (CHF), methanol fraction (MEF), and acetone extract of leaf (ACL) were examined for sedative activity.

### Chemicals

Diazepam was purchased from Square Pharmaceuticals Ltd., Bangladesh; thiopental sodium was purchased from Gonoshasthaya Pharmaceuticals Ltd., Bangladesh; 0.9 % sodium chloride solution (Normal saline) was purchased from Orion Infusion Ltd., Bangladesh and other reagents were of analytical grade.

### Animals

For the experiment *Swiss albino* mice of either sex, 4–5 weeks of age, weighing between 25–30 gm, were collected from the Animal Research Branch of the International Center for Diarrhoeal Disease and Research, Bangladesh (ICDDR, B). Animals were maintained under standard environmental conditions (temperature: (24.0 ± 1.0 °C), relative humidity: 55-65 % and 12 hrs light/12 hrs dark cycle) and had free access to feed and water *ad libitum*. The animals were acclimatized to laboratory condition for two weeks prior to experimentation. The number of protocol approval by the Ethics Committee of Jahangirnagar University, Dhaka, Bangladesh for the use of laboratory animals for the experiments.

### Drugs and Treatment

After reconstituted in distilled water all the extracts were administered to the mice at 100 and 200 mg/kg per orally by gavage. The water (5 ml/kg) was administered by gavage to the control group. All drugs, used as standard, were dissolved in 0.9 % saline and administered intraperitoneally (i.p.). Diazepam (2 mg/kg i.p.) was used as standard CNS depressant drug.

### Acute toxicity study

Mice were divided into control and test groups (n = 6). The test groups received the extract per orally at the doses of 500, 1000, 1500 and 2000 mg/kg. Then the animals were kept in separate cages and were allowed to food and *ad libitum*. The control group received the water. The animals were observed for possible behavioral changes, allergic reactions and mortality for the next 72 h [22].

### Neuropharmacological Activity

#### Thiopental sodium induced sleeping time test

The method described by Turner (1965) [23] was adopted to study the effect of the extracts of *Kaempferia galanga* on thiopental sodium induced sleeping time test. Test samples and control (n = 6) were administered orally but standard drug Diazepam (2 mg/kg) received intraperitonealy. Thirty minutes later, thiopental sodium (40 mg/kg, i.p.) was administered to each mouse to induce sleep. The animals were observed for the latent period (time between thiopental sodium administration to loss of righting reflex) and duration of sleep (time between the loss and recovery of reflex). Percentage of effect was calculated using the following formula:

$$ \mathbf{Effect}\ \left(\%\right)=\frac{\mathrm{Average}\ \mathrm{duration}\ \mathrm{of}\ \mathrm{loss}\ \mathrm{of}\ \mathrm{righting}\ \mathrm{reflex}\ \mathrm{in}\ \mathrm{the}\ \mathrm{test}\ \mathrm{group}}{\mathrm{Average}\ \mathrm{duration}\ \mathrm{of}\ \mathrm{loss}\ \mathrm{of}\ \mathrm{righting}\ \mathrm{reflex}\ \mathrm{in}\ \mathrm{the}\ \mathrm{control}\ \mathrm{group}}\times 100 $$

### Hole cross test

The method was adopted as described by Takagi *et al*. (1971) [24]. A partition was fixed in the middle of a cage having a size of 30 × 20 × 14 cm. A hole of 3 cm diameter was made at a height of 7.5 cm in the center of the cage. Mice were treated with control, standard or extract and were placed in one side of the cage. Then the number of passage of a mouse through the hole from one chamber to other was counted for a period of 3 min at 0, 30, 60, 90 and 120 min after the administration of the standard (i.p) and test drugs (p.o). Percentage inhibition of movements was calculated using the following formula:

$$ \mathbf{Movements}\ \mathbf{inhibition}\ \left(\%\right) = \frac{\mathrm{Mean}\ \mathrm{No}.\ \mathrm{of}\ \mathrm{movements}\ \left(\mathrm{control}\right) - \mathrm{Mean}\ \mathrm{No}.\ \mathrm{of}\ \mathrm{movements}\ \left(\mathrm{test}\right)}{\mathrm{Mean}\ \mathrm{No}.\ \mathrm{of}\ \mathrm{movements}\ \left(\mathrm{control}\right)}\times 100 $$

### Open field test

This experiment was carried out as described by Gupta *et al*. (1971) [25]. The mice were divided into control, standard, and test groups containing six mice each. Test group received *Kaempferia galanga* at the doses of 100 and 200 mg/kg (p.o.) whereas the control group received water (5 ml/kg, p.o.) and standard group received Diazepam (2 mg/kg, i.p.).The floor of an open field of half square meter was divided into a series of squares each alternatively colored black and white. The apparatus had a wall of 40 cm height. Mice were placed in the middle of the open field. Then the number of squares visited by the animals was counted for 3 min at 0, 30, 60, 90, and 120 min after the administration of the standard (i.p) and test drugs (p.o). Percentage inhibition of movements was calculated using the same formula used in hole cross test.

### Statistical Analysis

The statistical analysis for animal experiment was carried out using one-way ANOVA followed by Dunnett’s multiple comparisons. The results obtained were compared with the control group. *P* < 0.05 and *P* < 0.001 were considered to be statistically significant.

## Results

### Acute Toxicity

Oral administration of *Kaempferia galanga* at the doses of 500–2000 mg/kg did not produce any mortality or noticeable behavioral changes in mice within 72 hr observation period. Therefore, it can be suggested that *Kaempferia galanga* have low toxicity profile with LD_50_ higher than 2000 mg/kg.

### Neuropharmacological Activity

#### Thiopental sodium induced sleeping time test

All doses of the extracts produced a dose dependent decrease in onset of sleep and increase in duration of sleep. The results were found to be statistically significant (p < 0.05-0.001). In this test, ACR, PEF, CHF, MEF and ACL (200 mg/kg) showed maximum 276.65 %, 247.50 %, 358.55 %, 266.59 % and 171.11 % effect in duration of loss of righting reflex respectively, whereas the standard drug Diazepam (2 mg/kg) produced 231.42 % effect (Table 1).

**Table 1:** Effect of different extracts of *Kaempferia galanga* on thiopental sodium induced sleeping time test in mice

Group	Dose (mg/kg)	Onset of Sleep (minutes)	Duration of Sleep (minutes)	Percent effect
Control	5 ml/kg	2.06 ± 0.64	66.33 ± 8.04	100
Diazepam	2	1.36 ± 0.06	153.5 ± 11.53**	231.42
ACR	100	1.83 ± 0.38	83.33 ± 2.36	125.63
	200	1.67 ± 0.12	183.5 ± 21.40**	276.65
PEF	100	1.77 ± 0.18	105 ± 6.99	158.29
	200	1.89 ± 0.43	164.17 ± 17.61**	247.50
CHF	100	1.87 ± 0..25	97.67 ± 20.08	147.25
	200	1.28 ± 0.22	237.83 ± 8.09**	358.55
MEF	100	1.92 ± 0.23	95.17 ± 16.25	143.48
	200	1.51 ± 0.13	176.83 ± 24.57**	266.59
ACL	100	1.91 ± 0.24	69.5 ± 4.09	104.78
	200	1.41 ± 0.06	113.5 ± 15.65*	171.11

Control group received water 5 ml/kg (p.o), standard group received Diazepam 2 mg/kg body weight (i.p.), test groups ACR, PEF, CHF, MEF and ACL were treated with 100 and 200 mg/kg body weight of the extracts (p.o.) respectively. Values are mean ± SEM, (n = 6); * *p* < 0.05, **p < 0.001, *Dunnett t*-*test* as compared to control. ACR = Acetone extract of rhizome, PEF = Petroleum ether fraction of rhizome, **CHF** = Chloroform fraction of rhizome, **MEF** = Methanol fraction of rhizome and **ACL** = Acetone extract of leaf

### Hole cross test

The number of hole crossed from one chamber to another by mice of the control group was almost similar from 0 minute to 120 minutes (Table 2). Hole cross test of *Kaempferia galanga* showed significant decrease of movement from 30 to 120 minutes. The results were statistically significant (p < 0.05-0.001). In this test, maximum 93.93 %, 93.93 %, 93.3 %, 87.33 % and 95.09 % suppression of locomotor activity were exhibited with the ACR, PEF, CHF, MEF and ACL respectively. In this study Diazepam exhibited 71.70 % suppression.

**Table 2:** Effect of different extracts of *Kaempferia galanga* on hole cross test in mice

Group	Dose (mg/kg)	Number of movements (% of Number of movements inhibition)				
		0 min	30 min	60 min	90 min	120 min
Control	5 ml/kg	15.5 ± 1.84	14.33 ± 2.18	13.67 ± 1.83	10.5 ± 1.03	10 ± 1.41
Diazepam	2	15 ± 1.09	7.34 ± 0.76 (**48.78** %)	4.67 ± 0.61** (**65.83** %)	3.83 ± 0.75** (**63.53** %)	2.83 ± 0.59** (**71.70** %)
ACR	100	7.5 ± 2.36	2.83 ± 1.01** (**80.25** %)	2.33 ± 0.76** (**82.95** %)	1.83 ± 0.47** (**82.57** %)	3.33 ± 1.31* (**66.7** %)
	200	9.67 ± 1.76	1.83 ± 0.31** (**87.23** %)	0.83 ± 0.31** (**93.93** %)	1.5 ± 0.43** (**85.72** %)	1.67 ± 0.88** (**83.3** %)
PEF	100	11.33 ± 2.47	1.17 ± 0.31** (**91.83** %)	1.17 ± 0.40** (**91.44** %)	2 ± 0.82** (**80.95** %)	2.5 ± 1.05** (**75** %)
	200	8 ± 1.31	1 ± 0.26** (**93.02** %)	0.83 ± 0.47** (**93.93** %)	1.67 ± 0.61** (**84.09** %)	1.17 ± 0.60** (**88.3** %)
CHF	100	10.83 ± 0.65	4.83 ± 3.26* (**66.29** %)	2.5 ± 1.70** (**81.71** %)	3.67 ± 0.84** (**65.05** %)	2.17 ± 0.60** (**78.3** %)
	200	11.17 ± 3.09	4.5 ± 1.41* (**68.59** %)	1.83 ± 1.07** (**86.61** %)	1.5 ± 0.95** (**85.71** %)	0.67 ± 0.33** (**93.3** %)
MEF	100	8 ± 1.41	5.67 ± 2.82* (**60.43** %)	2 ± 1.06** (**85.37** %)	1.33 ± 0.71** (**87.33** %)	1.67 ± 0.61** (**83.3** %)
	200	11 ± 1.21	2.83 ± 1.19* (**80.25** %)	1.83 ± 0.54** (**86.61** %)	1.33 ± 0.49** (**87.33** %)	1.5 ± 0.50** (**85.00** %)
ACL	100	9.67 ± 1.99	2.5 ± 0.72** (**82.55** %)	2.67 ± 0.95** (**80.47** %)	2.5 ± 0.67** (**76.19** %)	1.83 ± 4.00** (**81.7** %)
	200	6.83 ± 2.01	1.33 ± 0.95** (**90.72** %)	0.67 ± 0.49** (**95.09** %)	1 ± 0.52** (**90.47** %)	0.5 ± 0.34** (**95.00** %)

Control group received water 5 ml/kg body weight (p.o.), standard group received Diazepam 2 mg/kg body weight (i.p.), test groups ACR, PEF, CHF, MEF and ACL were treated with 100 and 200 mg/kg body weight of the extracts (p.o.) respectively. Values are mean ± SEM, (n = 6); * *p* < 0.05, **p < 0.001, *Dunnett t*-*test* as compared to control. **ACR** = Acetone extract of rhizome, **PEF** = Petroleum ether fraction of rhizome, **CHF** = Chloroform fraction of rhizome, **MEF** = Methanol fraction of rhizome and **ACL** = Acetone extract of leaf

### Open field test

In the open field test *Kaempferia galanga* showed significant dose dependent decrease of movement from 30 to 120 minutes (Table 3). The results were statistically significant (p < 0.05-0.001). In this test, maximum 93.71 %, 92.83 %, 89.33 %, 89.56 % and 95.58 % suppression of locomotor activity were exhibited with the ACR, PEF, CHF, MEF and ACL respectively, whereas the standard drug Diazepam displayed 70.58 % suppression.

**Table 3:** Effect of different extracts of *Kaempferia galanga* on open field test in mice

Group	Dose (mg/kg)	Number of movements (% of Number of movements inhibition)				
		0 min	30 min	60 min	90 min	120 min
Control	5 ml/kg	112.83 ± 6.24	112.5 ± 5.67	103.33 ± 11.39	99 ± 3.95	90.67 ± 1.52
Diazepam	2	113 ± 2.62	57.83 ± 9.68* (**48.59** %)	46.67 ± 11.28* (**54.83** %)	37.83 ± 11.67** (**61.78** %)	26.67 ± 14.33** (**70.58** %)
ACR	100	64.83 ± 17.24	25.17 ± 12.4** (**77.62** %)	18 ± 4.76** (**82.58** %)	23.67 ± 7.54** (**76.09** %)	27 ± 6.76** (**70.22** %)
	200	91 ± 8.97	15.17 ± 4.73** (**86.52** %)	6.5 ± 2.38** (**93.71** %)	10.83 ± 3.58** (**89.06** %)	10.83 ± 3.98** (**88.05** %)
PEF	100	70.17 ± 11.86	14 ± 7.06** (**87.55** %)	12.17 ± 4.46** (**88.22** %)	11 ± 4.26** (**88.88** %)	17.33 ± 4.94** (**80.88** %)
	200	86.67 ± 11.14	13.83 ± 7.89** (**87.71** %)	9.5 ± 1.38** (**90.80** %)	9 ± 3.03** (**90.90** %)	6.5 ± 3.04** (**92.83** %)
CHF	100	90 ± 7.19	**31.5** ± 5.19** (**72.00** %)	24.33 ± 9.36** (**76.45** %)	25.83 ± 5.28** (**73.90** %)	15.33 ± 5.94** (**83.09** %)
	200	104.83 ± 8.18	12 ± 4.84** (**89.33** %)	11.67 ± 6.51** (**88.70** %)	11.67 ± 6.02** (**87.21** %)	13.83 ± 6.99** (**84.75** %)
MEF	100	84.17 ± 12.54	32.67 ± 17.79* (**70.96** %)	21.33 ± 11.97** (**79.36** %)	13.17 ± 3.50** (**86.69** %)	10.83 ± 7.06** (**88.05** %)
	200	90.83 ± 16.28	19.83 ± 7.66** (**82.37** %)	12 ± 3.71** (**88.38** %)	10.33 ± 3.67** (**89.56** %)	10.83 ± 3.98** (**88.05** %)
ACL	100	71.83 ± 22.56	44.83 ± 18.22* (**60.15** %)	26.83 ± 18.57** (**74.03** %)	11.33 ± 10.73** (**88.55** %)	6.33 ± 6.32** (**93.01** %)
	200	79.67 ± 11.34	31 ± 11.12* (**72.44** %)	16.17 ± 3.49** (**84.35** %)	6 ± 0.77** (**93.94** %)	4 ± 1.26** (**95.58** %)

Control group received water 5 ml/kg body weight (p.o.), standard group received Diazepam 2 mg/kg body weight (i.p.), test groups ACR, PEF, CHF, MEF and ACL were treated with 100 and 200 mg/kg body weight of the extracts (p.o.) respectively. Values are mean ± SEM, (n = 6); * *p* < 0.05, **p < 0.001, *Dunnett t*-*test* as compared to control. **ACR** = Acetone extract of rhizome, **PEF** = Petroleum ether fraction of rhizome, **CHF** = Chloroform fraction of rhizome, **MEF** = Methanol fraction of rhizome and **ACL** = Acetone extract of leaf.

## Discussion

The present study demonstrated that different extracts of *Kaempferia galanga* possess potent CNS depressant activity in thiopental sodium induced sleeping time, hole cross and open field models. No acute toxicity was observed after oral administration of *Kaempferia galanga* even at the dose of 2000 mg/kg in mice.

The most important step in evaluating drug action on the CNS is to observe the behavior of the test animals. Thiopental sodium induced hypnosis test revealed that all extracts, at the doses of 100 and 200 mg/kg body weight, dose dependently induced sleep at a rapid stage as compared to control and increased the duration of sleep. This is similar with the findings of Fujimori (1995) who proposed that the enhancement of barbital hypnosis is a good index of CNS depressant activity [26]. Substances that have CNS depressant activity either decrease the time for onset of sleep or prolong the duration of sleep or both. Another important step in evaluating drug action on CNS is to observe its effect on locomotors activity of the animal. The activity is a measure of the level of excitability of the CNS and this decrease may be closely related to sedation resulting from depression of the central nervous system [27, 28]. The extracts significantly decreased the locomotor activity as shown by the results of the hole cross and open field tests. The locomotor activity lowering effect was evident at the 2^nd^ observation (30 min) and continued up to 5^th^ observation period (120 min) (Table 2). Moreover, the validation of sedation was carried out by measuring external signs through hole-cross tests. Open field test showed that the depressing action of the extracts was also evident from the second observation period in the test animals at the doses of 100 and 200 mg/kg body weight. Maximum depressant effect was observed from 3^rd^ (60 min) to 5^th^ (120 min) observation period. The results were also dose dependent and statistically significant (Table 3). The reduction in sleep latency and increased total sleep time are classic parameters to relate the action of CNS depressants (Dandiya et al., 1959) [29]. Thus, considering that the fractions exerted its effects by decreasing sleep latency, increased total sleep duration by decreasing locomotion in the open field and hole cross test, the results indicate a sedative activity of *Kaempferia galanga.* Finally, this study suggests the possible CNS depressant activity of the different extracts of *Kaempferia galanga* on experimental animal models in dose dependent manner.

## Conclusion

Results of the present study indicate that all tested doses (100 and 200 mg/kg) of different extracts of *Kaempferia galanga* exhibited significant sedative effect. The effect is dose dependent, long lasting and statistically significant. Taking these findings into account it seems quite possible that *Kaempferia galanga* contains constituents with promising sedative activity. The traditional use of the plant in the treatment of stress, anxiety, depression etc. can be affirmed by this study. However, further studies are needed to isolate the pharmacological active compounds responsible for this activity.
